# A Qualitative Risk Assessment for Bluetongue Disease and African Horse Sickness: The Risk of Entry and Exposure at a UK Zoo

**DOI:** 10.3390/v14030502

**Published:** 2022-02-28

**Authors:** Elisabeth Nelson, William Thurston, Paul Pearce-Kelly, Hannah Jenkins, Mary Cameron, Simon Carpenter, Amanda Guthrie, Marion England

**Affiliations:** 1 Control of Infectious Diseases, London School of Hygiene and Tropical Medicine, London WC1E 7HT, UK; elisabeth.nelson1@alumni.lshtm.ac.uk (E.N.); mary.cameron@lshtm.ac.uk (M.C.); 2 Met Office, Exeter EX1 3PB, UK; william.thurston@metoffice.gov.uk; 3 Zoological Society of London, Regents Park, London NW1 4RY, UK; paul.pearce-kelly@zsl.org (P.P.-K.); hannah.jenkins@zsl.org (H.J.); amanda.guthrie@zsl.org (A.G.); 4 Species360, 7900 International Drive, Suite 300, Bloomington, MN 55425, USA; 5 School of Animal Rural and Environmental Sciences, Nottingham Trent University, Southwell NG25 0QF, UK; 6 The Pirbright Institute, Pirbright, Woking GU24 0NF, UK; simon.carpenter@pirbright.ac.uk

**Keywords:** bluetongue, African horse sickness, *Culicoides*, risk assessment, zoo

## Abstract

Bluetongue virus (BTV) and African horse sickness virus (AHSV) cause economically important diseases that are currently exotic to the United Kingdom (UK), but have significant potential for introduction and onward transmission. Given the susceptibility of animals kept in zoo collections to vector-borne diseases, a qualitative risk assessment for the introduction of BTV and AHSV to ZSL London Zoo was performed. Risk pathways for each virus were identified and assessed using published literature, animal import data and outputs from epidemiological models. Direct imports of infected animals, as well as wind-borne infected *Culicoides*, were considered as routes of incursion. The proximity of ongoing disease events in mainland Europe and proven capability of transmission to the UK places ZSL London Zoo at higher risk of BTV release and exposure (estimated as *low to medium*) than AHSV (estimated as *very low to low*). The recent long-range expansion of AHSV into Thailand from southern Africa highlights the need for vector competence studies of Palearctic *Culicoides* for AHSV to assess the risk of transmission in this region.

## 1. Introduction

Vector-borne diseases are an increasing global threat to the health of humans and animals with the spread of exotic pathogens facilitated by climate change, urbanization and extensive global travel and trade [1,2,3]. Historically, the United Kingdom (UK) has been largely protected from such pathogen incursion through its geographic isolation, temperate climate and socioeconomic development, but recent incursions of both novel vectors and vector-borne pathogens have occurred [4,5,6,7,8]. These events have triggered a series of exercises to identify future incursion risks and to highlight potential drivers of these events, including climate and land change [9,10,11,12,13]. 

Arboviruses are viruses that are transmitted by arthropods. Mosquitoes, ticks and biting midges can transmit medically important viruses that pose a risk to the UK [14]. Among emerging pathogens in northern Europe, two arboviruses of ruminants and deer transmitted by *Culicoides* biting midges (Diptera: Ceratopogonidae) have caused epidemics in the UK. Bluetongue virus (BTV: *Reoviridae*: *Orbivirus*) was detected in 2007 following an unprecedented outbreak in northern Europe that began in 2006 and was subsequently eradicated from the UK in the winter of 2007/2008, following a voluntary vaccination campaign [15,16,17]. Prior to this incursion, which involved a strain of BTV serotype 8 with a sub-Saharan origin, no *Culicoides*-borne arbovirus had ever been detected in the UK. Subsequently, Schmallenberg virus (SBV: *Peribunyaviridae: Orthobunyavirus*) was detected in the UK in 2011, in the same year as it was discovered in Germany, and remains endemic in the UK and northern Europe [18,19,20]. Furthermore, a broad diversity of additional strains of BTV have been transmitted successfully in northern Europe, but have not reached the UK, illustrating that this region has experienced a steep change in vulnerability to emergence of these arboviruses [21,22,23,24,25]. 

The impact of *Culicoides*-borne arboviruses in Europe is dependent upon both virulence, which is determined by the strain and species of virus and host-related factors [25,26]. A high proportion of emerging arboviruses that have been detected in northern Europe since 2006 have been largely ignored following initial assessment of pathogenicity (e.g., SBV [27]; BTV-25 [28]; BTV-14 [29]; BTV-6 [30]; BTV-11 [31]; and BTV-27 [32]). Other strains that cause more severe clinical signs in ruminants have triggered major responses with significant economic consequences, including culling, vaccination campaigns and long-term trade restrictions imposed to reduce spread (e.g., BTV-8 [26,33,34] and BTV-1 [33]). The UK currently imposes testing and control measures on ruminant imports from France (including the Mayenne region in the north), Spain, Luxembourg, Belgium and Germany, where BTV-8 has previously been detected [34]. The control strategy relies on vaccination, certification, post-import testing and monitoring of the disease situation in both Europe and internationally, and responsible sourcing of animals [35,36].

As a result of the emergence of BTV and SBV in northern Europe, this region is currently perceived to be at elevated risk of further incursions of *Culicoides*-borne arboviruses [25,37,38,39]. African horse sickness virus (AHSV), which is closely related to bluetongue virus [40], but causes disease in equine hosts, is the most cited example. It is one of the most lethal viral infections known in horses [41]. AHSV was isolated from pools of Palearctic species of *Culicoides* during the 1987–1991 outbreak of AHSV-4 in Spain, caused by the importation of infected zebra from Namibia [42,43]. While currently primarily circulating in sub-Saharan Africa [44,45], a strain of AHSV serotype 1 emerged unexpectedly in Thailand during 2020, which is suspected to have originated from importation of zebra from Africa [46]. This is the first time AHSV has occurred in Southeast Asia, and demonstrates the ability of the virus to be transmitted to new foci with no prior warning. 

Risk assessments for the importation of AHSV have been published from the point of view of live horse exports from the Republic of South Africa (RSA), including the use of pre-export quarantine [47] and additionally for northern Europe [48]. In addition, the UK has published an AHSV control strategy that outlines both likely routes of introduction and response [49]. While some routes of incursion of *Culicoides*-borne viruses are relatively well defined (e.g., movement of viraemic hosts and long-distance flight by infected *Culicoides*), the origin of several outbreaks into northern Europe remain unexplained, including the incursions of both BTV-8 [50,51] and SBV [52]. A key question has been the potential role of wildlife, including both the potential for wild species, moved as part of globalized trade, to carry unknown pathogens, as well as their susceptibility in the event of a new arbovirus outbreak. While both BTV and AHSV are generally restricted to ruminant and equine hosts, respectively, antibodies indicative of infection have been found for both viruses in a wide variety of additional vertebrate species, though the epidemiological relevance of these is poorly understood.

Zoological gardens are home to a wide variety of animals, many of which are of significant conservation concern. Animals kept in zoo collections are at risk of vector-borne diseases such as BTV and AHSV, and in some cases can be highly susceptible to severe manifestations of disease due to a lack of previous exposure to certain pathogens and increased potential exposure to the vectors [53]. Previous commentary has highlighted the potential impact of BTV on rare species in zoological collections, and has called for a detailed risk assessment of animal shipments from endemic regions [54]. Zoos, particularly in urban areas where stocking is dense, may facilitate cross-species disease spread by the presence of a diverse community of susceptible animals and through the inadvertent creation of attractive vector breeding habitats. ZSL (Zoological Society of London) London Zoo is situated in The Regent’s Park in the centre of London, an international hub and the largest city in the UK. In the event of a UK outbreak of BTV or AHSV, the surrounding farmland and wildlife could act as transmission reservoirs, enabling spill-over transmission to animals at the zoo. Of 49 zoos in northern Europe deemed at risk during the 2006 BTV outbreak, due to them being within 20km of a reported bluetongue outbreak, clinical disease was reported in 62 (6%) susceptible animals, with a case fatality rate of 69% [55]. A previous study has shown that the Palearctic vectors of BTV are present at ZSL London Zoo, with large numbers collected from light traps next to the Bactrian camels [56].

This study examines the potential routes of incursion of BTV and AHSV to the UK with specific reference to ZSL London Zoo. A qualitative risk assessment of potential importation pathways to ZSL London Zoo is conducted, and enables an understanding of the risk posed to animals in the zoo collection to inform preventative policies. 

## 2. Materials and Methods

### 2.1. Risk Assessment Methods

All potential entry routes of BTV and AHSV into the UK were considered and subsequently assessed within the context of zoological gardens. The specific risk questions were: (i) What is the probability that a susceptible animal at ZSL London Zoo can become infected with BTV?; and (ii) What is the probability that a susceptible animal at ZSL London Zoo can become infected with AHSV? The potential risk of incursion of exotic diseases can be assessed using the World Organization for Animal Health’s (OIE) Import Risk Analysis framework [57]. To answer the risk questions, entry and exposure assessments were conducted separately for each pathogen, according to the OIE Terrestrial Animal Health Code. Risk pathways for entry and exposure were identified, and the European Food Safety Authority’s (EFSA) qualitative probability definitions (Table 1) [58] were used to assign the level of risk associated with each step of each pathway, which were conditional probabilities. The overall qualitative probability of the risk pathway was then determined by combining the probabilities of the steps along the pathway and their weighted importance [59]. Strains and serotypes of the viruses were not considered separately, as underpinning data were not considered sufficient to allow differentiation. 

### 2.2. Risk Pathways

**Figure 1:**
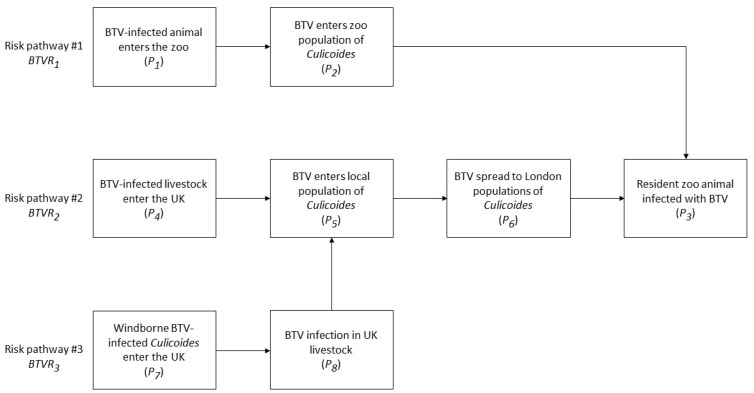
Risk pathways for the entry of BTV into ZSL London Zoo and exposure of susceptible resident animals.

**Figure 2:**
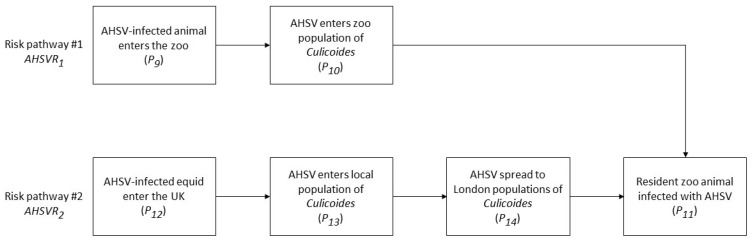
Risk pathways for the entry of AHSV into ZSL London Zoo and exposure of susceptible resident animals.

The risk pathways for entry of BTV and AHSV into ZSL London Zoo were identified. Entry and exposure assessments were conducted on three pathways for BTV and on two pathways for AHSV. Minor risk pathways were considered but rejected for assessment, as they were deemed to present a negligible risk (see Discussion). The risk pathways used in this risk assessment are given in Figure 1 and Figure 2. Within the wider context on BTV and AHVS incursion into the UK, risk pathways *BTVR*_2_, *BTVR*_3_ and *AHSVR*_2_ are pertinent across all regions, whereas *BTVR*_1_ and *AHSVR*_1_ are specific to certain locations and/or establishments, in this case zoological gardens. 

#### 2.2.1. Qualitative Probabilities for BTV Risk Pathways

##### Estimation of P_1_: Probability of BTV-Infected Animal Entering the Zoo

Direct entry of an infected animal from a BTV-endemic or -epidemic area is a potential route of virus incursion. This entry route is frequently cited as a potential source of new infections [51], and has previously been demonstrated as the source of BTV infection in ruminants in Poland [60]. The importation of BTV-infected cattle into the UK has occurred twice in the last five years, once in 2017 [61] and once in 2018 [62]. To estimate the risk associated with the importation of infected animals into a country or region, data are needed on the number and frequency of susceptible animals imported from BTV-endemic or -epidemic areas. The likelihood of an imported animal being infected is dependent on the prevalence of BTV infection in the country/region of origin, the length of viraemia, the vaccination status of the animal and the implementation of any control measures such as quarantine and pre- and post-import testing. 

Historically, zoological gardens in Europe have collected animals directly from the wild, with very little regard given to the pathogens they may be carrying [63]. This inevitably led to BTV-viraemic or seropositive animals entering zoos [64]. Modern zoos in Europe source most of their animals from other zoo collections and are subject to veterinary checks prior to transfer [65]. Screening for pathogens is carried out in accordance with EU and in-country legislation [66]. In the rare event that animals are collected from the wild, the European Association of Zoos and Aquaria guidelines require that zoos “carry out necessary veterinary screenings in accordance with official protocols” [67].

Between 2017–2020, ZSL London Zoo imported 52 animals from overseas (Supplementary Table S1) [68], but none of these animals were ruminants or considered to be susceptible to BTV infection, with the possible exception of a Sumatran tiger from Ebeltoft, Denmark in January 2019. While there is no evidence that feline species play an epidemiologically important role in outbreaks of BTV, antibodies have previously been reported in members of Felidae and Canidae [69], and it has been suggested they may become infected by oral ingestion of infected meat or through the bite of an infected vector [69,70,71]. During 2017, BTV was absent in all countries that exported animals to ZSL London Zoo. In 2018 and 2019, BTV was present in France, Canada and Germany [72], but only non-susceptible animals were imported from these countries to the zoo during this time (Supplementary Table S1). In response to the on-going outbreak of BTV in France and Germany, restriction zones have been set up, and voluntary vaccination for BTV-8 and BTV-4 is encouraged in the affected countries [73]. The UK currently requires all susceptible animals imported from France to be vaccinated against BTV [61]. 

If an animal is infected in the country of origin and then transferred to ZSL London Zoo, the length of the infectious period has to be considered. This is typically dictated by the length of viraemia in the host, however BTV has been isolated from the skin of sheep during the post-viraemic period at 42 days post-infection (d.p.i.) and 63 d.p.i. [74,75]. Sustained infection within the tissues is believed to be very rare, however, with several other studies failing to isolate the virus from the skin of post-viraemic cattle and sheep [74]. Additionally, it is possible to detect viral RNA by rt-PCR in the blood of infected ruminants after they are no longer infectious, but this is not considered to be epidemiologically significant [76]. The viraemic period is dependent on both host factors, such as species, breed, age and immunological status, as well as virus factors, such as strain [77]. Cattle are viraemic for longer than sheep, with virus isolation from the blood of cattle up to 60 d.p.i. and occasionally exceeding 100 d.p.i. [76]. Virus isolation from sheep has been observed up to 54 d.p.i. [78]. The OIE considers the viraemic period for ruminants to be up to 60 days, with a >99% probability of detectable viraemia ceasing by nine weeks in cattle [57,79]. This timeframe creates a plausible window for an infected animal to be imported into ZSL London Zoo. Given that antibodies have been found in other carnivore species, an infected tiger may be asymptomatic, increasing the chance of undetected viraemia prior to importation. However, given the provisions for the control and eradication of bluetongue in the EU outlined in EU Animal Health Law: Regulation (EU) 2016/429 [80] and Commission Delegated Regulation (EU) 2020/689 [81], including animal movement restrictions from affected areas to non-infected regions (that includes zoo animals), as well as strict border checks at both the UK border and within the zoo, the likelihood of an infected animal being undetected during the importation process is very low. 

##### Estimation of P_2_: Probability of BTV Entering *Culicoides* Population in the Zoo

Following importation of an infected animal, onward transmission of BTV would only be possible during the vector active season. In the UK, this is typically between early May and late October [82]. Within this period, vector populations fluctuate according to a bimodal pattern, with peak adult activity occurring in June and September [83]. Importations occurring outside of the vector active season would pose a negligible risk of onward transmission, whereas those occurring during the active season would be dependent on the activity of local populations of adult female *Culicoides*. In response to an incursion event, real-time localized trapping can be conducted through the UK *Culicoides* Reference Laboratory, although the implementation of a long-term national *Culicoides* surveillance network on livestock farms since 2006 permits accurate estimates of activity at any time throughout the year [84]. 

To assess the probability of onward transmission within ZSL London Zoo, data on the local vector population are required. Between June 2014–June 2015, a previous study collected 5768 *Culicoides* from ZSL London Zoo, comprising 25 different species [56]. The majority of the total catch (97.8%) constituted the putative vectors of BTV in northern Europe, *C. obsoletus*, *C. scoticus*, *C. dewulfi*, *C. chiopterus*, *C. pulicaris* and *C. punctatus* [25]. After bloodmeal analysis, *C. obsoletus/C. scoticus* specimens (the females of which cannot be morphologically distinguished) from ZSL London Zoo were found to have fed on alpaca/llama (*Vicugna pacos*/*Lama glama*) and Bactrian camels (*Camelus bactrianus*). Both these mammal species are susceptible to BTV infection.

The average length of viremia in a host which can infect a feeding *Culicoides* is 21 days, according to infection studies carrying out using *Culicoides sonorensis* on cattle and sheep [76], providing a reasonable window of time for an infected animal entering the zoo to be fed upon by multiple midges during viremia (assuming adult *Culicoides* activity). The species composition of *Culicoides* at the zoo reflects what is commonly found at livestock farms in northern Europe [85]. Previous BTV outbreaks in northern Europe have demonstrated that these *Culicoides* species are able to successfully transmit the virus within and between farms [4,86]. If an infected animal were to enter the zoo during the vector active season, it is reasonable that the virus would enter local populations of *Culicoides*. However, if the importation was to occur during the seasonal vector-free period, the risk of onwards transmission would be negligible.

##### Estimation of P_3_: Probability of Resident Zoo Animal Becoming Infected with BTV

Once BTV has entered a local population of *Culicoides*, susceptible animals in the surrounding area may be at risk of infection. The transmission rate of BTV is dependent on temperature, as this directly affects the extrinsic incubation period (EIP) of the virus as well as the activity of adult *Culicoides*. A recent study has identified temperature thresholds for *Culicoides* activity to be 4 °C in the autumn and 10 °C in the summer for populations in the south of England [87]. However, the temperature threshold for BTV replication within *Culicoides* is 12 °C [88], and therefore this is the most important threshold that must be reached for BTV transmission from *Culicoides* to occur.

Of the 414 individual mammals at ZSL London Zoo, 22 are highly likely to be susceptible to BTV infection (Table 2) [89]. During the 2006–2008 outbreak, clinical diseases were reported in 62 out of over 1000 susceptible animals held in European zoos, with a case fatality rate (CFR) of 69% in Bovidae [90]. This is considerably higher than the mean CFR seen in sheep and cattle during the outbreak, which were 22–41.5% in sheep and 0.22–51% in cattle [91,92,93]. Average daily temperatures in London exceed 12°C from approximately April to October, enabling both adult *Culicoides* activity and viral replication [94]. If BTV is present in local populations of *Culicoides* during this time, then susceptible zoo animals are at risk from BTV infection. 

The host-feeding preferences of *Culicoides* at ZSL London Zoo were discussed above, but it is important to note that a previous study found that the largest number of *Culicoides* collected at ZSL London Zoo were caught in the trap located near the Bactrian camels [56]. Bloodmeal analysis suggests that of all the susceptible animals in the zoo, the camels are at the highest risk of BTV infection. With the exception of wild birds, the zoo *Culicoides* population appears to be sustained primarily by zoo animals, which combined with the small geographic size of the zoo and the close proximity of the animals to each other, greatly increases the risk of transmission to susceptible zoo animals from infected *Culicoides*. 

##### Estimation of P_4_: Probability of BTV-Infected Livestock Entering the UK

In October 2017, post-import testing on a consignment of 32 cattle from France destined for two farms in England and two farms in Scotland identified BTV-8 positive animals [95]. BTV was again detected in late Autumn 2018 in French cattle imports [73]. The importations occurred in periods of low vector activity and strict movement restrictions were put in place on all detected farms, so no onward transmission occurred [96]. Between 2018–2020, a total of 102,515 BTV-susceptible animals were imported to the UK (Supplementary Table S2) [97]. Of these, 13,960 were imported from countries with BTV circulation. Given the previously mentioned length of viremia, an imported animal could be capable of onward transmission upon arrival in the presence of vector activity. Recently, Spain is the only country that has reported using BTV vaccines to the OIE, however, voluntary vaccination is encouraged in France and Germany, and restriction zones have been set up within those countries [73]. Vaccination is mandatory in Switzerland, and enforced in the export industry [73]. After the detection of the import cases in 2017, compliance issues with the vaccination status of cattle in the area of France were uncovered [95]. However, the UK initiates risk-based post-import checks of susceptible ruminants of EU-origin in accordance with Regulation (EU) 2017/625 [98], as well as documentary, identity and physical checks of animals from non-EU countries at border control posts [35]. The current BTV-8 outbreak in central and northern Europe is causing a wide range of non-specific clinical signs, and may therefore be difficult to differentiate from other common diseases. Cases are frequently mild or asymptomatic, with animals usually making a full recovery [22,75,99]. Without post-import testing, BTV could enter the UK and remain undetected for some time, facilitating onward transmission to the zoo.

In the UK, the two most commonly used real-time RT-PCR post-import tests are able to detect BTV in ruminants between 5 and 30 d.p.i. with the probability of detection ranging from 100% at peak viraemia down to 76% at 0–2 d.p.i. [100]. The probability of detecting a single positive individual reduces significantly in the early stages of infection, if multiple samples are pooled. The tests were designed for detection of serotypes 1–24, but may be unable to detect the more recently discovered “atypical” serotypes of BTV [100]. Differentiation between infected and vaccinated animals (DIVA) is an issue when using serological tests to detect BTV infection, as there are currently no commercially available DIVA-compliant vaccines [99]. However, the routine use of RT-PCR assays for post-import testing in the UK negates this problem. There is the potential for detection of BTV RNA in the blood of sheep up to nine days post-vaccination [101], and for up to three days in the blood of cattle [102]. This could present a potential onward risk of transmission if the vaccine used was a modified-live virus vaccine (MLV), which has previously been shown to replicate in *Culicoides* [103]. 

##### Estimation of P_5_: Probability of BTV Entering Local *Culicoides* Populations

The 2006–2009 BTV-8 outbreak in northern Europe demonstrated the vectorial capacity of Palearctic *Culicoides* species, namely members of the *C. obsoletus* and *C. pulicaris* complexes. In laboratory tests, *C. obsoletus* from different geographic regions of the UK were found to have BTV infection rates ranging from 0.4–7.4%, and *C. pulicaris* specimens collected from Keele, UK, were found to have a 13% infection rate [104]. Some populations of Palearctic species could reach infection rates of up to 26% using membrane and pad-feeding, exceeding those recorded for BTV’s putative vector in Africa, *Culicoides imicola* [104]. Given the large populations of *Culicoides* vectors throughout the UK, substantial transmission in the absence of control measures remains possible during the vector active season. Livestock density and land use has been linked to *Culicoides* abundance, with larger populations in areas with higher livestock density [82,105]. BTV spreads to *Culicoides* more effectively in warmer conditions when populations peak due to more rapid life cycles. However, BTV can persist in a latent phase in infected *Culicoides* for long periods in cold temperatures, resuming replication once the temperatures increase [106].

Biting rates of vector *Culicoides* on livestock can be extremely high, and have been observed to be in excess of 150 bites per minute on sheep [107]. If a BTV-positive livestock import occurs during the vector active season, and average daily temperatures are >12 °C, it is likely that BTV could enter local populations of *Culicoides*. 

##### Estimation of P_6_: Probability of Spread of BTV to London *Culicoides* Populations

Midge dispersal has been found to be the principal mode of transmission of BTV between farms [108]. This phenomenon of midge dispersal is referred to as a ‘stepping stone effect,’ in which a sequence of short-range infections result in what appears to be a long-distance transmission [109]. During the 2006–2009 BTV-8 outbreak in northern Europe, 54% of new cases occurred over distances up to 5 km, 92% over distances up to 31 km and only 2% over distances greater than 31 km [109]. If infected livestock were imported to a farm in the UK, this ‘stepping stone effect’ could potentially carry the infection to London *Culicoides* populations, with proximity of the initial farm to London determining the time scale. Additionally, the *Culicoides* species composition on farms surrounding London is suitable for BTV transmission, with vector species present on farms in Hertfordshire, Essex, Kent, Berkshire and Surrey (M. England, unpublished data) [82]. 

The proximity of susceptible livestock to London may be a limiting factor for *Culicoides* dispersal. The density of cattle and sheep is low in the London area [110,111], but there is a relatively high density of goats in some parts of Greater London, with 2–25 animals per km^2^ [112]. The nearest livestock holdings to London Zoo are two city farms (1.93 km and 3 km distance from ZSL London Zoo) that have small holdings of cattle, sheep and goats. Further small holdings that are open to the public extend outwards from central London and are all within approximately 5 km of each other. This creates a network of livestock holdings across London that are well within the transmission range of 31 km observed during the 2006–2009 BTV outbreak [109].

##### Estimation of P_7_: Probability of Windborne BTV-Infected *Culicoides* Entering the UK

**Figure 3:**
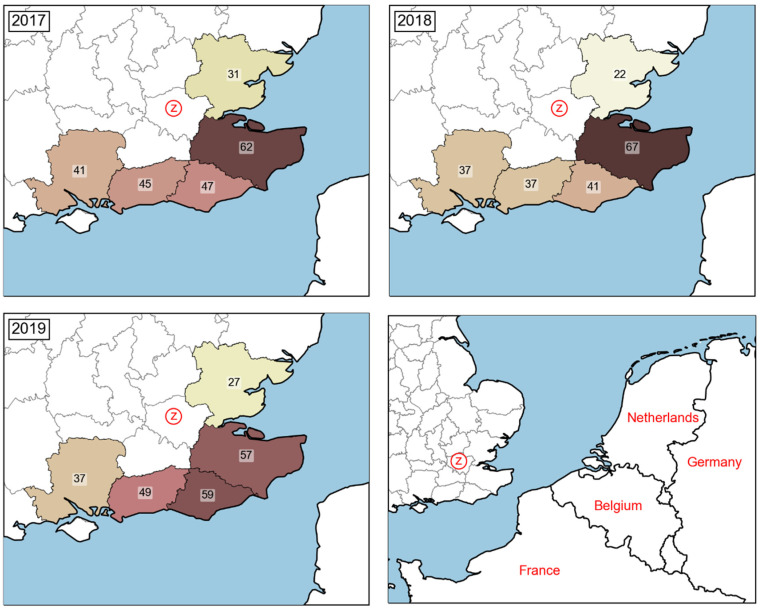
NAME-modelled *Culicoides* incursions from all European sources to the UK. Total number of potential incursions in 2017, 2018 and 2019 [114]. ZSL London Zoo is marked on the map in red.

During the 2006–2008 northern Europe outbreak of BTV-8, it was proposed that incursion into the UK occurred through long-distance wind dispersal of infected *Culicoides* from continental Europe [113]. The small body size of *Culicoides* (1–3 mm in length) enables their semi-passive dispersion over great distances by wind [114]. The UK Met Office’s Numerical Atmospheric-dispersion Modelling Environment (NAME) [115] models the release, spread and removal from the atmosphere of windblown midges, by analysing meteorological data and data on *Culicoides* populations [114]. According to routine outputs from NAME model runs, performed during the vector active season to estimate the potential for windborne *Culicoides* incursion to the UK [38,116,117], there were approximately 226 potential incursions of windblown midges from continental Europe into UK coastal counties near London in 2017, 204 in 2018 and 229 in 2019 (Figure 3). The UK shares many species of *Culicoides* with northern Europe, including the putative vectors of BTV. A previous study found *C. obsoletus* accounted for 83% of *Culicoides* trapped in nine EU countries between 2007–2013 [85]. Therefore, competent vectors are likely present along the coast of continental Europe. Based upon OIE reports, it is believed that BTV was present in France and Germany from 2017 to 2020, and present in Belgium in 2019–2020 [73]. It is likely that infected *Culicoides* would survive after entry into the UK, particularly since incursions would likely occur during a period of high vector activity, enabling them to be caught by the wind. 

##### Estimation of P_8_: Probability of BTV-Infection in Native UK Livestock following Windborne Incursion of Infected *Culicoides*

The greatest risk for onward transmission of BTV after an incursion of infected *Culicoides* occurs in areas with high livestock densities close to the coast. Comparing data on livestock density with the incursions shown in Figure 3, East Sussex and Kent are at the greatest risk for onward transmission. The rate of transmission is highest on cattle-only farms, followed by sheep-only farms, and lowest on mixed farms [116]. All five counties that experience incursions as determined by the NAME model (Figure 3), have similar densities of cattle and goats, but East Sussex and Kent have higher densities of sheep, potentially increasing their risk for infection [110,111,112]. In the absence of vaccination, modelling has shown that there is a high chance of disease spread beyond the initial site of incursion [118]. Additionally, modelled incursions occurring in September result in smaller outbreaks with less geographical spread than incursions occurring in May. Incursions occurring earlier in the year have more time for disease spread, taking full advantage of the adult *Culicoides* active season [118]. 

#### 2.2.2. Qualitative Probabilities for AHSV Risk Pathways

##### Estimation of P_9_: Probability of AHSV-Infected Animal Entering the Zoo

The recent outbreak of AHSV in Thailand is hypothesised to be the result of the importation of an infected equid from an AHSV-endemic country. This shows that despite having appropriate precautions in place, as required by the OIE, it may still be possible for infected equids to enter a country, either illegally or through incorrect certification. To improve disease control and to prevent fraud, in 2018 the UK introduced new equine identification regulations making it a legal requirement for all equids to be microchipped, with fixed penalty fines for noncompliance [117]. All horses imported into the UK from other countries (including the EU) must be accompanied by a health certificate issued after physical inspection of the horse [119]. This certificate also requires that the horse is entering the UK from a region that has been free from AHSV for two years. 

In 2019, ZSL London Zoo housed two plains zebra (*Equus quagga chapmani*) (Table 3) which can act as reservoir hosts for AHSV, driving its distribution and persistence in endemic regions of Africa [40,44]. The export of AHSV-infected zebra from Namibia to Spain in 1987 caused an outbreak that lasted for three years [120]. Zebra are viraemic for up to 40 days, so it is reasonable that an asymptomatic zebra could have a transmissible infection upon entry into the UK [41]. AHSV has never been reported in any of the countries from which animals were imported to ZSL London Zoo over the last three years, and none of the imported animals were equids (Supplementary Table S1). Additionally, export countries have not reported the use of any vaccine to OIE, and the use of live attenuated vaccines is not permitted in AHSV-free regions [121]. Susceptible equids undergo pre- and post-import testing in the UK if arriving from an AHSV-endemic or seasonally endemic area. They are also required to isolate in an AHSV-free area or vector-proof housing for a period of up to 40 days prior to importation with appropriate serological and/or antigen testing [57]. Post-import diagnostic tests used are highly sensitive and specific, so are likely to correctly identify the presence of virus or antibodies. However, pre- and post-import testing for AHSV in the UK is only carried out on equids, yet asymptomatic infections can occur in carnivores, in particular big cats [69]. The domestic dog is the only non-equid species known to exhibit severe disease, and it has been suggested that natural infection could occur via a non-oral, vector-mediated route [122]. Therefore, it may be possible for animals other than equids to introduce AHSV to the UK. 

##### Estimation of P_10_: Probability of AHSV Entering *Culicoides* Populations in the Zoo

The primary vector of AHSV is *C. imicola*, which is found in high abundance across most of AHSV’s known range [40]. However, during the 1987–1991 outbreak in Spain, AHSV was isolated from pooled samples containing *C. obsoletus* and *C. pulicaris*, and lacking *C. imicola* [42]. In Portugal, it was postulated that transmission was driven by *C. imicola*, and the coinciding high abundance of *C. obsoletus* and *C. pulicaris* allowed the virus to enter these species [123]. These findings support the theory that *C. obsoletus* and *C. pulicaris* could act as vectors in the absence of *C. imicola*, as is the case with BTV in northern Europe [124]. Prevalence of AHSV infection in *Culicoides* is often less than 10%, so transmission relies on high abundance and biting pressure [40]. *Culicoides obsoletus* in the zoo have been shown to be non-specific opportunistic feeders, and thus have the potential to feed on an AHSV-infected animal following importation into the zoo. 

##### Estimation of P_11_: Probability of a Resident Zoo Animal Becoming Infected with AHSV

In 2019, there were 15 individual animals considered susceptible to AHSV kept in the ZSL London Zoo collection (Table 3) [89]. Previous studies have shown antibodies to AHSV in dromedary camels [125,126], but the potential for infection in Bactrian camels is unknown. For the purposes of this risk assessment, we are assuming the Bactrian camels at ZSL London Zoo present a susceptible population. The small geographic size of the zoo and the proximity of the animals would permit transmission by *Culicoides* in the event of an incursion. During the 1987 outbreak in Spain, widespread transmission occurred to other local equids, and resulted in an outbreak encompassing three countries [127]. The transmission rate is related to seasonal variation in *Culicoides* population abundance and to the extrinsic incubation period (EIP) of the virus, which in turn is dependent on temperature. The summer months in London are likely the only months capable of supporting transmission. Laboratory studies on the bluetongue vector, *Culicoides sonorensis*, have shown that replication of AHSV ceases at ≤15 °C [128]. Average daily temperatures in London typically exceed 15 °C during the months of June, July and August [129], and therefore an AHSV-infected animal imported during this time could present a risk of onward transmission. In South Africa, average daily temperatures exceed 15 °C from October to May [130], which permits transmission throughout a significant proportion of the year. Assuming vector competence of *C. obsoletus* group species and/or *C. pulicaris* group species to AHSV, it is possible that transmission between an imported equid and susceptible zoo animals could occur if the import were to occur between the months of June and August. 

##### Estimation of P_12_: Probability of AHSV-Infected Equid Entering the UK

Between 2018–2020, the UK imported 16,380 equids from EU countries, and between 2018–2019, 4,254 equids from non-EU countries (Supplementary Table S3) [97]. In 2018 and 2019, AHSV was absent in all countries that exported animals to the UK. In March 2020, an outbreak of AHS began in Thailand, but there have been no reports during 2020 from any of the other export countries. No vaccination use was reported to OIE by any of the export countries between 2018–2020 [131]. In EU countries, AHSV has been a notifiable disease since December 1982, and EU countries are required to have contingency plans in operation with restriction and surveillance zones [80]. Outside the EU, most non-endemic countries require import testing and quarantine of equids and similar action plans if an infection is detected. In endemic countries, which neighbor a few of the export countries (such as Morocco and Tunisia), live attenuated vaccines are routinely used and movement restrictions are employed in the event of an outbreak [132]. Once in the UK, the probability an infected equid passes border checks is very low, due to strict pre- and post-import testing required, as previously mentioned. The OIE Terrestrial Code defines the infective period as 40 days for domestic horses [57], while donkeys are viraemic up to 17 days [133]. A range of highly sensitive and specific RT-PCR and rRT-PCR assays are used by AHSV diagnostic laboratories [134]. These tests can detect all nine known serotypes of AHSV. The OIE lays out a number of conditions that must be met prior to export from an infected country, including that horses are not permitted to travel within 40 days of receiving a vaccination [57]. In South Africa, outbreaks of AHSV have been caused by a reversion to virulence or reassortment of AHSV live attenuated vaccines, which can be spread by *Culicoides* [135].

Horses commonly exhibit severe symptoms which would likely be detected during routine veterinary checks at border posts. However, horses from endemic regions may present mild or sub-clinical infection due to frequent exposure to natural infection and/or vaccination. A previous study focusing on competition thoroughbred horses has assessed the risk of an undetected AHSV-infected horse being exported from both low-risk and endemic areas of South Africa [47]. It was estimated that with post-import testing and post-arrival quarantine in place, the risk was equivalent to one undetected infected horse in every 2.2 million horses exported from low-risk areas. This increased 15 to 17 times if the horse came from an endemic area. The risk would likely vary greatly depending on the type/breed of horse being imported, its prior exposure and its vaccination status.

##### Estimation of P_13_: Probability of AHSV Entering Local UK *Culicoides* Populations

As previously mentioned, vector competence for AHSV of the Palearctic species *C. obsoletus* and *C. pulicaris* has been suggested. These species are widespread across the UK in high abundance, comprising between 93.5–97% of specimens caught on farms, with traps in some locations catching thousands of specimens in a single night [136]. Equine holdings have shown similar *Culicoides* species composition and abundance [137,138]. Given the large populations of potential vectors found in the UK and the higher rate of AHSV infection observed in *Culicoides* populations compared to infection rates for BTV, it is likely AHSV circulation could occur. Studies in the UK, France and the Netherlands determined *C. obsoletus* and *C. pulicaris* bite horses, so onward transmission would likely occur after initial importation of an infected equid, assuming the adult vectors were active and temperatures were sufficient for viral replication within the vector [139,140,141]. The destination of imported horses has been found to cluster in south-east England, where temperatures may be sufficient to enable transmission during the summer months [142]. 

##### Estimation of P_14_: Probability of Spread of AHSV to London *Culicoides* Populations

Given suitable conditions for AHSV circulation within UK *Culicoides* populations, transmission would likely follow a similar ‘stepping stone’ pattern to BTV, with small jumps between equine holdings. The species composition in London is likely suitable for AHSV transmission, given that studies at ZSL London Zoo caught mainly members of the *C. obsoletus* and *C. pulicaris* complexes, albeit in lower numbers than those typically seen on farms [56]. Unfortunately, there is limited data on the distribution and numbers of horses and other equids in the UK. The potential spatio-temporal transmission rates of AHSV in Great Britain have been modelled previously using ambient temperatures during the year, seasonal abundance of *Culicoides*, and the distribution of other hosts [139]. The model found the patterns of transmission were mainly influenced by the abundance of *Culicoides*, the distribution of horses and the presence of non-susceptible hosts (sheep and cattle). Previous estimates of horse density across Great Britain indicates low density in London, which could limit transmission potential [139].

## 3. Results

### 3.1. BTV Risk Pathways

The lack of susceptible imported animals from countries with BTV transmission over the last few years, the low probability for an infected animal to pass border checks in its country of origin and the UK, as well as veterinary inspection at the zoo, greatly reduce the probability of a BTV-infected animal entering the zoo. However, given the possibility for asymptomatic animals to be imported the probability, *P*_1_, is classified as *very low*. This probability is based on the assumptions that tigers may be able to carry infection undetected, there is no opportunity for exposure during transit and that EU and Canadian border checks are being correctly adhered to. If an infected animal is imported into ZSL London Zoo, the suitable species composition and feeding preferences of the zoo *Culicoides* populations render the probability of BTV-infected *Culicoides* in the zoo, *P*_2_, as *medium* during the vector active season. If, however, the importation occurred outside the vector active season or when temperatures are below that required for viral replication, *P*_2_ would be considered *negligible*. The probability of a zoo animal becoming infected, *P*_3_, if BTV-infected *Culicoides* are present in the zoo, is *high* to *very high*, given the availability of susceptible zoo animals kept in close proximity to one another (Table 2) and the demonstrated host feeding preferences of the *Culicoides* populations in the zoo. The probability of BTV-infected livestock entering the UK, *P*_4_, is *very low*. Border checks and post-import testing currently appear to be working well, however the frequency of imports of susceptible animals from countries with ongoing BTV circulation (Supplementary Table S2) presents an ongoing low-level risk of entry of an infected animal. Additionally, there is uncertainty around the ability to detect atypical BTV serotypes. Given the large abundance of members of the *C. obsoletus* and *C. pulicaris* complexes throughout the UK, their proven vectorial capacity and their association with livestock, the probability that BTV enters native *Culicoides* populations, *P*_5_, from an imported infected animal is *medium* during the vector active seasonal and *negligible* during the seasonal vector-free period. The probability of spread of BTV to London *Culicoides* populations, *P*_6_, from a primary import site is *low*. While an infection could plausibly spread to London from infected farms through midge dispersal, and competent vector species are present in London, the risk is reduced by the low densities of cattle and sheep in and around Greater London.

Windborne incursion of *Culicoides* to UK coastal counties near London is predicted to occur over 200 times each year (Figure 3). Case reports of BTV in 2020 have only occurred beyond 150 km of the coast of the UK, although positive cases of BTV-8 have been reported in northern France (the closest to the UK are in the Mayenne region of northwest France) [143]. Therefore, the probability, *P*_7_, is *low* during the vector active season and *negligible* during the seasonal vector-free period. The probability of BTV-infection in native livestock following a windborne incursion of an infected *Culicoides*, *P*_8_, is *medium*. The presence of unvaccinated cattle, sheep and goats in coastal counties with frequent incursions throughout the year presents a highly susceptible population. However, the associated dependence on midge survival after importation for successful onward transmission limits this probability to *medium*.

When the above probabilities are combined, the overall probability for risk pathway *BTVR*_1_ is *low*, for risk pathway *BTVR*_2_ is *low* to *medium* and for risk pathway *BTVR*_3_ is *medium*. These probabilities are calculated for the vector active season. During the seasonal vector-free period, all risk pathways would present *negligible* probabilities due to the nature of BTV being a vector-borne virus. The qualitative risk probabilities are summarized in Table 4.

### 3.2. AHSV Risk Pathways

The probability of an AHSV-infected animal entering the zoo, *P*_9_, is *very low*, given that AHSV has never been reported in any of the export countries and no equids were imported into the zoo over the last three years (Supplementary Table S1). The risk is not considered *negligible* due to the potential for asymptomatic infection in imported non-equids. The probability of AHSV entering zoo populations of *Culicoides*, *P*_10_, following the importation of an infected animal is *medium* (during the vector active season, *negligible* during the seasonal vector-free period), given the existence of populations of potential AHSV vectors in the zoo and their proven feeding on a wide range of hosts. The vector competence of northern European *Culicoides* for AHSV is unknown, so this probability estimate assumes that members of the *Avaritia* subgenus, *C. pulicaris* and *C. punctatus* are competent vectors. This is based on their ability to act as vectors for BTV, and the vector status of *C. imicola* for both BTV and AHSV in endemic regions. The probability of a resident zoo animal becoming infected with AHSV, *P*_11_, is *medium*, due to the availability of susceptible zoo animals (Table 3) and demonstrated feeding preferences of *Culicoides* populations in the zoo and across northern Europe. This assumes that AHSV can replicate and disseminate to the salivary glands successfully in UK *Culicoides* under suitable environmental conditions.

The probability of an AHSV-infected equid entering the UK, *P*_12_, is *very low*, due to the absence of the disease in all but one of the countries that have exported equids to the UK in the last two years (Supplementary Table S3), as well as the strict control measures in place both pre- and post-import into the UK. However, the large number of susceptible equids imported into the UK every year and the global nature of horse travel does create a non-negligible risk, as proven in 2020 by the AHSV outbreak in Thailand. The probability of AHSV entering local *Culicoides* populations, *P*_13_, is *low* to *medium*, given the large populations of potentially competent *Culicoides* known to be present around equine facilities in the UK and the suitability of summer temperatures at the destinations of the majority of imported equids. This would, of course, be *negligible* during the seasonal vector-free period. The probability of AHSV spreading to London *Culicoides* populations, *P*_14_, is *very low*, given the limited host distribution in the immediate London area.

When the above probabilities are combined, the overall probability for risk pathway *AHSVR*_1_ is very *low* and for risk pathway *AHSVR*_2_ is *very low* to *low* during the vector active season. During the seasonal vector-free period, all risk pathways would present *negligible* probabilities. The qualitative risk probabilities are summarized in Table 5.

## 4. Discussion

The risk pathways describing the probable entry and incursion routes of BTV and AHSV have been identified and qualitatively assessed. For both viruses, the pathways are very similar, largely due to their shared *Culicoides* vectors. The major divergence in risk between the two diseases is associated with their different geographical distributions, as BTV is already established in northern Europe. The BTV outbreaks recorded in northern Europe and the UK demonstrate the virus’ ability to replicate in temperate conditions, be transmitted by Palearctic *Culicoides* species, and spread rapidly through naïve populations of livestock [4,113]. Its current persistence in northern Europe puts the UK at continual risk of re-introduction. In contrast, AHSV has never been reported in temperate regions, and there have only been a few incursions into southern Europe [120,140]. Very little is known about its potential to spread in Palearctic *Culicoides*, but its geographic distance from the UK lessens the overall risk of introduction. The current status of scientific knowledge and distribution of AHSV is remarkably similar to that of BTV before its breakthrough to northern Europe. Additionally, an outbreak of AHSV in the UK would disrupt an industry (horse racing, performance sports and recreation combined) that is worth £7 billion a year to the UK economy [141]. Therefore, while the greatest risk of the two viruses to ZSL London Zoo is currently posed by BTV (Table 4), the potential impact of an AHSV outbreak cannot be ignored. 

The most likely pathway of BTV introduction into the UK and on to ZSL London Zoo is from long-distance windborne incursion of infected *Culicoides* from France, Belgium or the Netherlands. This pathway, *BTVR*_3_, has been estimated to present a *medium* level of risk to the UK (Table 4). Evidence suggests such windborne incursions were responsible for the 2007 BTV-8 outbreak in the UK [109,114]. The continued presence of the disease in countries from which models show hundreds of potential windborne *Culicoides* incursions annually (Figure 3) results in this pathway currently presenting a constant risk during the vector active season. The other two risk pathways considered here for BTV (*BTVR*_1_ and *BTVR*_2_) both have the potential to occur and have been estimated to present a risk of *low* and *low* to *medium*, respectively (Table 4). If either of these routes of incursion coincide with the vector active season in the UK, onward transmission is possible. However, the likelihood that an infected zoo animal or livestock import would pass both pre-import testing in its country of origin and post-import testing within the UK is very low. While infected cattle have departed from France undetected, the rapid identification of these cases after arrival in the UK highlights the effectiveness of the UK’s post-import surveillance [95]. Additionally, the zoo has not imported any animals that are known to be susceptible to BTV in the past few years (Supplementary Table S1) [68].

The introduction and onward transmission of AHSV presents a lower risk to the UK than BTV, through either an infected animal imported directly to the zoo (risk pathway #1, *AHSV*_1_, Figure 2) or an infected equid imported to the UK (risk pathway #2, *AHSV*_2_, Figure 2)). The severe consequences associated with clinical disease usually seen in horses, have forced strict pre- and post-import checks that greatly reduce the likelihood that an infection would go undetected. The potential for transmission in non-equid hosts is poorly understood, and this risk assessment has attempted to capture this uncertainty by considering the possible role of big cats as asymptomatic carriers. Additionally, the racehorse industry is a global network, and as has been seen recently in Thailand, the potential for new outbreak foci cannot be excluded. For this reason, the two pathways considered for AHSV were not deemed to be *negligible* (except for during the seasonal vector-free period). For both AHSV and BTV, the control measures in place to stop disease spread play a significant role in reducing the risk of introduction and exposure. 

### 4.1. Key Assumptions

For the purposes of this risk assessment, different serotypes and strains of BTV and AHSV were not considered separately. There are phenotypic differences between strains of BTV, which can affect detection potential. Currently, there are 24 serotypes of BTV that are transmitted by *Culicoides* biting midges and are largely similar in clinical presentation [144]. Since 2008, further “atypical” strains of BTV have been discovered, such as BTV-25 which does not cause clinical signs in goats and only mild clinical signs in sheep [145]. Indeed, BTV-26 does not replicate experimentally within *Culicoides sonorensis*, although infection studies with European species of *Culicoides* have yet to be conducted [146]. Instead, there is evidence that BTV-26 can be transmitted via direct contact between goats [146]. As well as heterogeneous geographical distribution, the differential transmission pathways of BTV serotypes have a direct impact on the risk of disease introduction to the UK, as well as the ability for pre- and post-import detection. Further studies to better understand the transmission pathways of atypical BTV serotypes are required to improve future risk assessments. There are nine serotypes of AHSV, which are variously distributed throughout central and sub-Saharan Africa [147]. It is unknown whether there are similarly “atypical” serotypes of AHSV circulating, and this presents further uncertainty when trying to assess incursion risk.

From previous studies, the vector active season at ZSL London Zoo was found to begin in late April and end in late October/early November [56]. For this risk assessment, it has been assumed that all vector species are equally active throughout the season, and temporal variation in risk throughout the active season has not been considered. However, a previous study sampled *Culicoides* at 12 sites across the UK and found that *Culicoides* on farms exhibited bimodal seasonality, with populations peaking in April/May and then again in September/October [83]. There was variation between the sites, but lower abundance was noted across all sites in June. *Culicoides punctatus* and *C. pulicaris* emerged earliest, and were continually caught later than other species, demonstrating a longer active season compared to members of the subgenus *Avaritia*. These variations would directly impact the risk of BTV and AHSV introduction, as transmission would be less likely in periods with lower population abundance. Therefore, the greatest risk for onward transmission would occur in April/May and September/October, but within-year variation is not reflected in this risk assessment. 

Another assumption for this risk assessment was that UK *Culicoides* species are capable of transmitting AHSV. This assumption is based on their ability to transmit the closely-related bluetongue virus and the shared vector competence of *C. imicola* for BTV and AHSV in endemic regions [148]. The length of the extrinsic incubation period and temperature replication thresholds for AHSV in the UK was estimated based on laboratory work performed on *C. sonorensis* [106,128]. There is clearly a need to determine the vector competence of Palaearctic species of *Culicoides* for AHSV to more accurately assess risk to the UK, and to aid preparedness and response in the event of an outbreak. 

The Sumatran tiger was assumed to be susceptible to both BTV and AHSV based on evidence of antibodies in other big cats and carnivores [69,90]. There are substantial levels of uncertainty around this assumption, given that it is unknown whether carnivores can be infected by vector feeding or solely through oral transmission, and whether onward transmission would then be possible. It is unclear whether an infection would be asymptomatic or have a clinical presentation, and what the length of the incubation and viraemic periods would be. Without specific evidence of infection in tigers, their possible role in transmission of both AHSV and BTV can only be assumed based on evidence in related species. To understand the role of different animals in AHSV transmission, it would be useful to assess the impact, if any, of AHSV on zoo animals in endemic regions, and the seroprevalence within zoos across a range of species.

The reliability of OIE country reports for BTV is unknown, given countries that are under restriction and protection zones may not report all disease events. The probability estimates given in this risk assessment are based entirely on reports available through EU reporting channels and those reported publicly by the OIE. The risk to the UK is highly dependent on the proximity of outbreaks to the coast of northern Europe, and continual monitoring of *Culicoides* vector activity within the UK and potential airborne incursion events as modelled by NAME are required to understand real-time disease risk throughout the year. 

With regards to livestock and equine imports to the UK, there will always be variation between years, so the precise risk will vary accordingly between and within years. In this risk assessment, outputs from the NAME model were used to determine windborne incursions to the UK (Figure 3). The nature of the model itself carries inherent uncertainty, since it uses historical NAME outputs to determine future risk. The NAME model predicts the windborne transport of midges across water, but once over land is unable to determine flight behaviour and where they will land. Therefore, it was assumed that areas with higher livestock densities would attract more midges, but what triggers *Culicoides* to land in certain locations is unknown. The survival rates and number of midges arriving is also unknown, as the model treats them as particles transported by the wind for a fixed length of time. Studies that investigate how *Culicoides* fly over land, and what drives them to land after passive wind transport, would also be useful to increase the predictability of the NAME model, and therefore increase accuracy when estimating risk from wind-borne incursions.

The low densities of both livestock and equine populations within the Greater London area present a potential break in the stepping-stone effect of transmission. The majority of *Culicoides* collections in the UK have taken place on farms in rural or semi-rural areas, and whilst *Culicoides* vector populations are known to be present within ZSL London Zoo, trapping within the Greater London area has not been conducted. Transmission potential of BTV between small holdings in urban and semi-urban areas needs to be assessed by investigating host and vector populations and their interactions in these areas. This would provide a more complete understanding of whether susceptible animals within ZSL London Zoo are protected from *Culicoides*-borne viruses by urban barriers. The presence of small livestock holdings, largely in the form of petting zoos, across London provide a potential network for disease transmission. Data on the distribution of horses across London is patchy but the most visible equids in London are working horses. There are eight stables that house the approximately 110 working horses of the Metropolitan Police Mounted Branch, seven of which are within Greater London [149]. Additionally, the City of London Police has a mounted branch which has stables close to St. Paul’s Cathedral [150]. These horses are moved out of the city once a week for rest and space. There are 211 horses of the Household Cavalry housed at Hyde Park [151], and additional high value horses kept in the Royal Mews at Buckingham Palace [152]. The daily movement of these horses around London, and their movements into and out of the city could potentially facilitate AHSV transmission in the event of an outbreak.

Due to the nature of the industry, there are countless small- and large-scale movements of horses around the UK every day from localized riding for pleasure to long-distance transport for organized equestrian events such as polo. Evidence from previous outbreaks in Spain and Thailand has shown that horse owners respond to an AHSV incursion by moving their horses away from the affected areas, which inadvertently facilitates more widespread transmission [S. Carpenter, personal communication]. These movements are not necessarily illegal, but there is an increase in the frequency of movements at the start of an outbreak that makes the disease harder to contain and control. 

### 4.2. Negligible Risk Pathways

The risk pathways assessed in this study were determined to be the most likely pathways for the incursion of BTV and AHSV into the UK, with the potential to cause onward transmission to an animal in ZSL London Zoo. The selection of these pathways was based upon available research on the elements of each pathway and current scientific understanding of the diseases. Additional risk pathways were identified, but considered to pose a negligible risk (including during the vector active season) at this time. These are outlined below. 

For BTV, it has been suggested that disease introduction could occur through the importation of infected midges with cargo, such as cut flowers. Initially, the 2006–2009 BTV-8 outbreak in northern Europe was thought to have originated via this pathway, since initial cases occurred in Maastricht, an international plant trading hub [25]. This was later thought not be the case following the discovery of earlier infections on farms nearer to Belgium [153]. However, a previous study surveyed international ships arriving in Qinhuangdao Port, China during the summer of 2003, and found that 29 of 70 ships inspected contained live midges, including species of *Culicoides* [154]. The UK imports 17% of Kenya’s flower exports (Kenya is a BTV- and AHSV-endemic country), creating an opportunity for this pathway to occur [155]. Flowers are grown in specific areas near to Nairobi airport, from where they are shipped directly via aeroplane at low temperatures to the UK, and then directly on to supermarkets [J. Stokes, personal communication]. Given the lack of susceptible livestock at either end of this pathway, as well as the conditions of travel, the risk of incursion from this pathway is considered negligible. 

Another risk pathway for BTV is the potential importation of infected germplasm. Transmission is possible via either frozen or chilled germplasm, and it has been proposed that frozen bull semen from 2007 caused the resurgence of BTV-8 in France in 2015 [156]. The risk of disease importation to the UK is currently negligible via this pathway, given the testing measures in place at semen collection centres and the strict legislation surrounding the importation of specimens from restricted areas. 

Finally, a possible risk pathway for AHSV incursion occurs through the long-distance spread of infected midges via wind movements. In Africa, winds transporting infected *Culicoides* from endemic regions have caused outbreaks in naïve equid populations in non-endemic areas [23]. The maximum possible distance for dispersal has been postulated as 700 km over water, or 150 km over land [148]. The risk to the UK from this route is therefore negligible, due to the absence of AHSV within this geographical range. 

## 5. Conclusions

Bluetongue virus and African horse sickness virus are two closely related *Culicoides*-borne viruses, which have immense economic consequences and disrupt global trade. After careful analysis of their risk of introduction to the UK and onward transmission to ZSL London Zoo through the assessment of the most likely risk pathways, BTV was found to pose a greater threat, but the uncertainty surrounding AHSV may underestimate (or overestimate) the risk from the pathways assessed in this study. Overall, the probability of BTV infecting a zoo animal in ZSL London Zoo was determined as *low* to *medium*, with the most likely route of infection being through the windborne introduction of infected *Culicoides* from mainland Europe followed by onward transmission and spread to the zoo. The probability of a zoo animal becoming infected with AHSV was determined to be *very low* to *low*, according to the risk pathways assessed.

To mitigate against the threat of BTV or AHSV introduction into the UK and the zoo collection, there are several strategies that can be adopted. Stringent post-import testing on ruminants and equids for BTV and AHSV respectively should continue, and potentially expand to encompass the previously mentioned species found to have antibodies when imported from countries with known disease. When importing animals from high-risk areas appropriate quarantine periods should be adhered to, with the recommendation to use vector-proof housing during this period. Additionally, if animals were to be imported during the winter when *Culicoides* adult activity is minimum, this would reduce the risk of onward transmission. In the event of an incursion of BTV to the UK, all susceptible animals should be vaccinated with a serotype-specific vaccine. Vaccination against AHSV would depend on amendments to current licensing, and would only be advised in the event of an outbreak due to the possibility of reversion to virulence of live vaccine strains, and the implications for longer term AHS-free status. Surveillance of *Culicoides* populations within the zoo and within Greater London is recommended to monitor seasonal activity patterns and to detect any changes in vector abundance. In the event of an outbreak of either BTV or AHSV in the UK, reducing vector-host contact through vector-protective housing and restricting outdoor access to periods of low vector activity (midday) would greatly reduce the risk of infection.

## Figures and Tables

**Table 1:** Definitions of qualitative probability categories [58].

Risk Probability	Definition
Negligible	Event is so rare that it does not merit consideration
Very low	Event is very rare but cannot be excluded
Low	Event is rare but does occur
Medium	Event occurs regularly
High	Event occurs very often
Very high	Event occurs almost certainly

**Table 2:** ZSL London Zoo animals at risk of BTV infection in 2019 [89].

Scientific Name	Common Name	Total No. of Animals
*Camelus bactrianus*	Bactrian camel	2
*Muntiacus reevesi*	Chinese muntjac	2
*Giraffa camelopardalis*	Giraffe	3
*Okapia johnstoni*	Okapi	3
*Capra hircus*	Nigerian goat	4
*Capra hircus*	West African pygmy goat	3
*Cephalophus natalensis*	Red forest duiker	2
*Lama glama*	Llama	2
*Vicugna pacos*	Alpaca	1
**Total**		22

**Table 3:** ZSL London Zoo animals at risk of AHSV infection in 2019 [89] [H. Jenkins, personal communication].

Scientific Name	Common Name	Total
*Lycaon pictus*	African hunting dog	7
*Equus asinus*	Donkey	2
*Equus quagga burchelli*	Burchell’s zebra	2
*Equus quagga chapmani*	Chapman’s zebra	2
*Camelus bactrianus domestic*	Bactrian camel	2
**Total**		15

**Table 4:** Qualitative probability estimates for BTV risk parameters and pathways during the vector active season.

Probability	Qualitative Probability
BTV-infected animal enters the zoo (*P*_1_)	*Very low*
BTV enters zoo population of *Culicoides* (*P*_2_)	*Medium*
Resident zoo animal infected with BTV (*P*_3_)	*High* to *very high*
BTV-infected livestock enters the UK (*P*_4_)	*Very low*
BTV enters local population of *Culicoides* (*P*_5_)	*Medium*
BTV spread to London populations of *Culicoides* (*P*_6_)	*Low*
Windborne BTV-infected *Culicoides* enter the UK (*P*_7_)	*Low*
BTV infection in UK livestock (*P*_8_)	*Medium*
**Risk pathway #1, *BTVR*_1_ (*P*_1_, *P*_2_, *P*_3_)**	***Low***
**Risk pathway #2, *BTVR*_2_ (*P*_4_, *P*_5_, *P*_6_, *P*_3_)**	***Low* to *medium***
**Risk pathway #3, *BTVR*_3_ (*P*_7_, *P*_8_, *P*_5_, *P*_6_, *P*_3_)**	***Medium***

**Table 5:** Qualitative probability estimates for AHSV risk parameters and pathways during the vector active season.

Probability	Qualitative Probability
AHSV-infected animal enters the zoo (*P*_9_)	*Very low*
AHSV enters zoo population of *Culicoides* (*P*_10_)	*Medium*
Resident zoo animal infected with AHSV (*P*_11_)	*Medium*
AHSV-infected equid enters the UK (*P*_12_)	*Very low*
AHSV enters local population of *Culicoides* (*P*_13_)	*Low* to *medium*
AHSV spread to London populations of *Culicoides* (*P*_14_)	*Very low*
**Risk pathway #1, *AHSVR*_1_ (*P*_9_, *P*_10_, *P*_11_)**	***Very low***
**Risk pathway #2, *AHSVR*_2_ (*P*_12_, *P*_13_, *P*_14_, *P*_11_)**	***Very low* to *low***

## Data Availability

Data is contained within the article or supplementary material.

## Supplementary Materials

The following supporting information can be downloaded at: www.mdpi.com/article/10.3390/v14030502/s1, Supplementary Table S1: Imports of animals to ZSL London Zoo from outside of the UK (including Jersey). Supplementary Table S2: Ruminant imports from EU countries January 2018–July 2020. Supplementary Table S3: Imported equids from non-EU countries from January 2018–December 2019.
