# Supplementary material for: A Qualitative Risk Assessment for Bluetongue Disease and African Horse Sickness: The Risk of Entry and Exposure at a UK Zoo
Source: Viruses. 2022 Feb 28;14(3):502. doi: 10.3390/v14030502 (PMC8950286; doi:10.3390/v14030502)
Supplement: Supplementary file 1 [file viruses-14-00502-s001.zip › Supplementary Table S1.pdf]

**Supplementary Table S1: Imports of animals to ZSL London Zoo from outside of the UK (including Jersey).** From January 2017-December 2019 [69].

| Country | City | Animal Common Name | Animal Scientific Name | No. of Animals | Date of import |
|---|---|---|---|---|---|
| Austria | Vienna | Charco Palma pupfish | *Cyprinodon veronicae* | 1 | 14/12/2017 |
| | | Killifish | *Aphanius saldae* | 1 | 14/12/2017 |
| | | La Palma Pupfish, Cachorrito de Charco Palmal | *Cyprinodon longidorsalis* | 1 | 14/12/2017 |
| | | Potosi pupfish | *Cyprinodon alvarezi* | 1 | 14/12/2017 |
| Canada | Toronto | Big-headed turtle | *Platystemon megacephalum* | 4 | 25/09/2018 |
| | | Malagasy cichlid | *Ptychochromic insolitus* | 3 | 03/10/2019 |
| | | Panchax | *Panchypanchax amoulti* | 1 | 03/10/2019 |
| | | Zonobe rainbowfish | *Rheocies vatosoa* | 1 | 03/10/2019 |
| Czech Republic | Praha | Moholi bushbaby | *Galago moholi* | 1 | 12/04/2017 |
| | Olomouc | Southern tamandua | *Tamandua tetradactyla* | 1 | 07/09/2017 |
| Denmark | Ebeltoft | Sumatran tiger | *Panthera tigris sumatrae* | 1 | 28/01/2019 |
| France | Lille Zo | Grey parrot | *Psittacus erithacus* | 4 | 24/01/2018 |
| Germany | Hamburg | Dwarf mongoose | *Helogale parvula undulatus* | 2 | 19/09/2017 |
| | Frankfurt | Grey slender loris | *Loris lydekkerianus grandis* | 1 | 24/01/2018 |
| | Koln | Abdim's stork | *Ciconia abdimii* | 1 | 26/06/2018 |
| Netherlands | Rotterdam | Hyacinth macaw | *Anodorhynchus hyacinthinus* | 1 | 28/07/2018 |
| Switzerland | Zurich | Galapagos tortoise | *Chelonoidis nigra* | 8 | 28/02/2017 |
| | | Panther chameleon | *Furcifer pardalis* | 2 | 19/12/2019 |
| UK | Jersey | Round Island skink | *Leiolopisma telfarii* | 16 | 21/07/2017 |
| | | Giant jumping rat | *Hypogeomys antimena* | 1 | 19/12/2018 |
