# Supplementary material for: A Qualitative Risk Assessment for Bluetongue Disease and African Horse Sickness: The Risk of Entry and Exposure at a UK Zoo
Source: Viruses. 2022 Feb 28;14(3):502. doi: 10.3390/v14030502 (PMC8950286; doi:10.3390/v14030502)
Supplement: Supplementary file 1 [file viruses-14-00502-s001.zip › Supplementary Table S2.pdf]

Supplementary Table S2: Ruminant imports from EU countries January 2018-July 2020. No imports occurred from non-EU countries in these years [97]. Countries and years with reported BTV circulation are marked in red.

**Country**	***Species***	**2018 (No. of animals)**	**2019 (No. of animals)**	**2020 (No. of animals)**
Austria	*Bos taurus*	74	0	0
Belgium	*Bos taurus*	957	1621	33
Czech Republic	*Bos spp*	1	0	0
	Bos taurus	0	1	0
Denmark	*Bos taurus*	2962	3858	0
France	*Bos taurus*	1228	230	18
Germany	*Bos spp*	2	0	0
	Bos taurus	4683	6019	32
Ireland	*Bison spp*	0	2	0
	Bos taurus	29838	38930	6
Italy	*Bos taurus*	11	4	0
	Bubalus bubalis	48	25	0
Luxembourg	*Bos taurus*	601	629	0
Norway	*Bos taurus*	0	8	0
Poland	*Bos taurus*	0	1	0
Spain	*Bos taurus*	6	0	0
Sweden	*Bos taurus*	140	349	0
Netherlands	*Bos taurus*	7810	2394	0
**Total**		**48355**	**54071**	**89**
