# Supplementary material for: A Qualitative Risk Assessment for Bluetongue Disease and African Horse Sickness: The Risk of Entry and Exposure at a UK Zoo
Source: Viruses. 2022 Feb 28;14(3):502. doi: 10.3390/v14030502 (PMC8950286; doi:10.3390/v14030502)
Supplement: Supplementary file 1 [file viruses-14-00502-s001.zip › Supplementary Table S3.pdf]

Supplementary Table S3: Imported equids from non-EU countries from January 2018-December 2019. An additional 16,307 horses, 44 donkeys and 29 mules were imported from EU countries from January 2018-July 2020 [97].

**Country of Origin**	**2018 (No. of Horses)**	**2019 (No. of Horses)**
Argentina	423	430
Australia	93	124
Bahrain	24	35
Barbados	2	0
Canada	35	52
Chile	8	4
China	14	6
Hong Kong	41	31
Iceland	5	18
Indonesia	10	0
Israel	2	5
Japan	13	18
Jordan	0	6
Korea, Republic of	1	2
Kuwait	8	18
Malaysia	2	6
Mauritius	142	87
Morocco	16	45
New Zealand	27	34
Oman	43	50
Peru	0	6
Qatar	35	37
Russian Federation	47	21
Saudi Arabia	3	16
Serbia	0	1
Singapore	3	9
Thailand	1	5
Tunisia	0	7
Turkey	0	3
Ukraine	4	17
United Arab Emirates	484	600
United States	559	498
Uruguay	16	2
**Total**	**2061**	**2193**
